# Interactive and automated application of virtual microscopy

**DOI:** 10.1186/1746-1596-6-S1-S10

**Published:** 2011-03-30

**Authors:** Klaus Kayser, Jürgen Görtler, Stephan Borkenfeld, Gian Kayser

**Affiliations:** 1 UICC-TPCC, Charite, Berlin, Germany; 2 IAT, Heidelberg, Germany; 3 IBM, Mainz, Germany; 4 Institute of Pathology, University Freiburg, Freiburg, Germany

## Abstract

Virtual microscopy can be applied in an interactive and an automated manner. Interactive application is performed in close association to conventional microscopy. It includes image standardization suitable to the performance of an individual pathologist such as image colorization, white color balance, or individual adjusted brightness. The steering commands have to include selection of wanted magnification, easy navigation, notification, and simple measurements (distances, areas). The display of the histological image should be adjusted to the physical limits of the human eye, which are determined by a view angle of approximately 35 seconds. A more sophisticated performance should include acoustic commands that replace the corresponding visual commands. Automated virtual microscopy includes so-called microscopy assistants which can be defined similar to the developed assistants in computer based editing systems (Microsoft Word, etc.). These include an automated image standardization and correction algorithms that excludes images of poor quality (for example uni-colored or out-of-focus images), an automated selection of the most appropriate field of view, an automated selection of the best magnification, and finally proposals of the most probable diagnosis. A quality control of the final diagnosis, and feedback to the laboratory determine the proposed system. The already developed tools of such a system are described in detail, as well as the results of first trials. In order to enhance the speed of such a system, and to allow further user-independent development a distributed implementation probably based upon Grid technology seems to be appropriate. The advantages of such a system as well as the present pathology environment and its expectations will be discussed in detail.

## Introduction

Virtual microscopy is, by definition, the work with completely digitized glass slides, i.e. virtual slides [1-5]. This does include the viewing of histological images, their interpretation, the procedures of deriving of a diagnosis, and the transfer of the evaluated diagnosis to the clinician, who usually treats the patient [6]. All mandatory additional procedures such as the patients history, radiological images, or data derived from previous examinations are included too. Thus, virtual microscopy is the diagnostic work with digitized data that contribute to the diagnosis [7,8]. The basic knowledge how to work with, and how potential errors can be avoided (or at least minimized) has been collected by development, trials, and quality assurance investigations on telepathology [9-12]. In fact, the development of telepathology, which is the diagnostic work on histological images at a distance [13-15] is a characteristic example how medical application and newly developed technologies interact [16], and how aims in focus changed due to the limits of medical use. Giving an example: The primarily aim of telepathology was its application in frozen section services, and quite a number pioneers who investigated in this application can be named [15]. This method has been called on-line telepathology, or remote control telepathology [14]. The second method, to apply telepathology in an off-line mode, or to give experts the opportunity to view (and evaluate) difficult cases was of minor interest to the majority of pathologists, and only a few took attention and investigated in this method [14] in the early times of telepathology. About ten years later the situation has changed completely, and the outstanding majority of pathologists use telepathology for expert consultation purposes [2,9,11,13,17].

What are the reasons of the changes in telepathology interest?

They are two folds: 1) The technology could not completely fulfil the medical demands: A well trained technician has to prepare the frozen section glass slides, and a well trained doctor has to sample the specimen with the most efficient diagnostic findings in terms of a histological image. No technical assistants are available in the described human performance. In addition, the quality of cutting frozen section mainly depends upon the frequency how often it is performed, and in hospitals with numerous frozen sections a local pathologist is usually “on board”. Thus, most of the calculations whether telepathology should be implemented or not resulted in a cost efficiency computation, and in a comparison of tissue transportation time against savings of time in the surgical theatre [13]. These ideas might direct certain local decision in favour to implement a telepathology system; they are, however, no solid reason to unavoidably spread telepathology [13,15].

2) The main problem of medical communication, namely the existence of a firm and user independent standard has been solved by introducing the so-called internet [2,5,10,11,14,18-20]. Its main impact on medical communication was the opening of a network that was accessible for all partners who want to participate [14]. It can be considered as mandatory condition to perform expert consultation from “any place of the world” to “any expert”. As a result, expert consultation is now-a-days the main application of telepathology [14].

What is the present position and what are the expectations of virtual microscopy?

## Basic considerations

The performance of virtual microscopy is basically independent from any workflow of the pathology laboratory [3,5,17,21]. It is a work in a completely digitized world, of course with human interference [4]. The human interference can be limited to the work with a conventional microscope, i.e., to changes in magnification, of illumination, of focus, or to navigation through the slide without any further computerized actions. This performance is called interactive microscopy [5]. It can, in addition, supported by computerized assistants that perform the navigation, magnification, etc. in comparison to so-called assistants in programs such as “word”, “excel”, etc. The final stage of such assistants would be a diagnosis assistant that calculates the probability of diagnoses that can be derived from the specific virtual slide [3]. This procedure is called automated virtual pathology.

What are the specificities and features of interactive virtual microscopy?

## Interactive virtual microscopy

**Figure 1:**
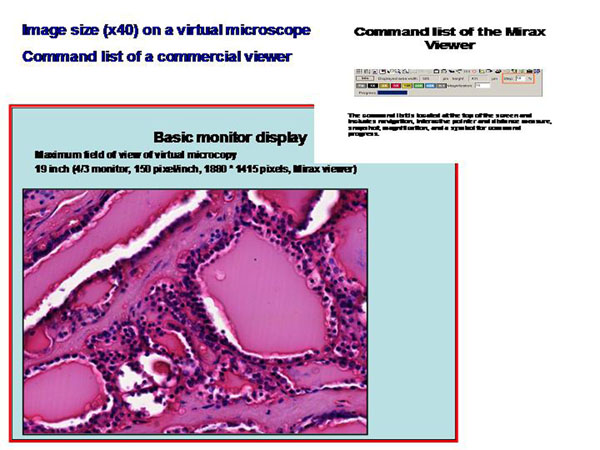
Screenshot of a virtual slide in an analogue size as presented by a commercially available viewer (Mirax). The command list of working with the virtual slide is separately shown at the right upper corner <kayser-interactive_fig_1.jpg>

Interactive virtual microscopy possesses certain specificities that are not known in conventional microscopy despite the basic performance is similar. Similar or even identical are the commands of navigation, magnification, focussing, or illumination. Most of the commercial available systems present with a similar arrangement of these commands on the screen, indicating that they expect a pathologist’s performance comparable to that on a conventional microscope, as demonstrated in <figure 1>. There can, in addition, several tools be implemented that work only in a digital environment. These include the selection of field of view, contemporary display of overlaid images or labels, of images of different magnification, artificial colouring, and implementation of sound. These additional tools want either to induce a more comfortable performance of slide viewing, or to increase the diagnostic security, or both [15,22].

## Automated virtual microscopy

Automated virtual microscopy tries to transfer some or even all work of the pathologist to a computerized system that performs computations on image quality, spatial distribution of image information content including the selection of fields of view, the evaluation of the most likely diagnosis, and statistical calculations of quality assurance. Although no fully developed automated diagnosis system is available at present, theoretical considerations and trials performed on still images indicate that automated virtual microscopy is not a fiction [3,21,23,24]. In addition, the development of virtual slide scanners point in the direction of implementing enhanced image analysis software with sophisticated image feature classifiers. The final aim is obviously an automated virtual microscope with features that elevate the pathologists work to a higher, more attractive level.

Developed tools include the analysis of image quality for both interactive and automated virtual microscopy [5]. They take into account the limited field of vision and the subjective color sensation of humans who have to view TV screens in interactive microscopy, and color and illumination correction, as well as contrast enhancing methods in automated virtual microscopy [7]. An automated assessment of the required magnification in measuring image features and the automated selection of the most interesting fields of view have to be added. Different algorithms have been described to be applied in virtual microscopy [4]. In principle, two different approaches can be distinguished:

The diagnostic work of a pathologist is based upon the recognition and classification of image information [21]. Clinical information such as age and sex of the patient, duration of symptoms have to be taken into account too, especially in diagnostic difficult cases. The computerization of this process can focus on the receiver’s side (pathologist), or on the sender’s side (image), or on both [21]. Approaches that focus on the pathologist’s side have to manage two problems: 1) To analyze the broad variety of images that belong to the same diagnosed disease.

2) To translate the different ways of diagnostics that themselves depend upon the disease to be classified (for example, the diagnostic procedure of a pathologist in classifying a chronic inflammatory significantly differs from that of classifying a cancer!).

The advantages of the described classic approach are relatively “simple” statistics, unbiased material, and direct comparison with the gold standard (conventional diagnosis).

Approaches to analyze image information “at its source” and in the first step independently from the receiver (pathologist) require precise definition of “image information”, standardized images, and detailed knowledge of the “diagnosis transformation algorithms” [25]. The final result should be a “clinical useful diagnosis”, and not an expression of entropy, diffusion terms, etc. [25]. An additional disadvantage is the missing interactivity of a pathologist who cannot control whether the system works correct (or not) prior to the final result [26]. The advantage is the implementation of a fully automated diagnostic system which is controlled by the pathologist only at its end stage [26].

## Implementation and expectations

Implementation and expectations differ for the described systems. Interactive virtual microscopy is mainly bound to the scanners installed in an institute. All commercially available scanners are provided with an own viewer that primarily allows the viewing of the digitized images with inbuilt functions that are derived from the work with conventional microscopes. Some vendors have included bar code recognition and retrieval function that allow the communication with a hospital information system (HIS). The mandatory standards (HL7, Picture Archiving and Communication System (PACS)) are not fully developed, and have to be modified for microscopic and gross images. Several working groups are working on this standardization, and it can be expected that a common standard will be available in the near future. Two institutes of pathology located in The Netherlands and in Sweden already use interactive virtual pathology in daily routine diagnostics, partly contemporary with conventional microscopy.

The application of automated virtual microscopy in routine surgical pathology has not been reported to our knowledge. At present, the investigations focus on tests and implementation of the necessary standards, especially PACS and DICOM. An additional focus is the automated identification of the areas of interests [26,27]. Several research teams are working on reliable and practical (minimum computation time) solutions [26,27]. It can be expected that first implementations will occur within the next two years similar to the implementation of evaluation of image quality and automated feature extraction. Another three to four years might pass until this new diagnostic technique will reach the stage of ß testing. Thus, pathologists who are eager to work with this new technology will probably have to wait for another five to six years; however, most probably, not for a longer time.

## Competing interests

The authors declare that they have no competing interests.
